# The preferred IT sources and tools of Iranian people for accessing health information

**DOI:** 10.1186/s12889-023-16334-y

**Published:** 2023-10-12

**Authors:** Farzad Salmanizadeh, Nazanin Jannati, Leila Ahmadian, Yunes Jahani, Mohsen Balouchi, Reza Khajouei

**Affiliations:** 1 Department of Health Information Sciences, Faculty of Management and Medical Information Science, Kerman University of Medical Sciences, Kerman, Iran; 2 Modeling in Health Research Center, Institute for Futures Studies in Health, Kerman University of Medical Sciences, Kerman, Iran

**Keywords:** Health information seeking, Health information needs, Information technology, Information sources

## Abstract

**Introduction:**

People need health information to maintain their health. Despite the variety of sources and tools for providing health information, there is little evidence about Iranian people's preferences in using these sources and tools. The objective of this study was to identify the preferred health information sources, tools, and methods for presenting health information in these tools.

**Methods:**

This national survey was conducted among a sample of 4000 Iranian people between April and September 2021. The data was collected using a valid and reliable questionnaire (α = 0.86) consisting of four sections: participants' demographic information, current sources of obtaining health information, preferred information technology (IT) tools for accessing health information, and the method of presenting this information. Linear regression was used to investigate the relationship between demographic factors and other questions.

**Results:**

The participants received health information mostly from the "Internet" (3.62), "family or friends" (3.43), "social networks" (3.41), "specific websites" (3.41), and "mobile apps" (3.27). "Social networks" (3.67), Internet "websites" (3.56), and "mobile apps" (3.50) were the most suitable tools for receiving health information. The participants preferred the presentation of health information in the form of "Images" (3.85), "educational videos" (3.69), and "texts" (3.53). Age, education, and marital status had a significant relationship with most of the preferred information sources, tools, and information presentation methods (*p* < 0.05).

**Conclusion:**

The results of this study showed that Iranian people are more active information seekers than passive ones compared to a decade ago. The preferred sources and tools identified in this research can be used by healthcare planners and policy-makers in Iran and other developing countries to design and develop IT interventions that meet people's needs. Improving access to the Internet, social networks, and mobile apps and providing health information via images, educational videos, and texts on these platforms enhance access to the information people need.

**Supplementary Information:**

The online version contains supplementary material available at 10.1186/s12889-023-16334-y.

## Introduction

Receiving proper health information is one of the crucial needs of people that resulted in the development of health information systems in different countries [1]. Health information includes a wide range of information about the prevention and primary treatment of diseases, which is one of the main concerns of people and the main component of health promotion [2]. Providing information to patients improves health outcomes, such as reducing stress, increasing satisfaction, increasing disease control, rising compliance with treatment regimens, and improving communication between patients and healthcare providers [3]. According to the reports of the World Health Organization (WHO), people's access to health information is vital for improving health systems in different societies [4] and can effectively support disease prevention and treatment processes [5]. Despite the importance of obtaining information, people cannot easily meet their information needs. To create fair access to health information and improve health communication, some governments, including the US government, are planning to improve health outcomes for people, by proposing programs such as the "Healthy People 2030" [6] and establishing special laws (such as the American Recovery Reinvestment Act of 2009) to [7]. The advancement of Information and Communications Technology (ICT) enabled the development of health applications and the accessibility of health websites to the public and this allowed laypeople to learn, and know more about disease prevention, self-care, and health information seeking [8]. Studies have shown that health information seeking enhances individual knowledge, lifestyle habits [9], patient communication with service providers, and medical decision-making [10] and reduces stress and negative emotions [11]. It also helps people use each other's experiences to manage their stress [12].

Identifying the different sources through which people obtain health information helps to determine the role of each source in information-seeking behavior. Various studies have been conducted on health information sources in recent years, mainly in developed countries [13–17]. Different kinds of information sources are used to seek health information and support different information needs of people. These sources include newspapers [18], magazines [19], television [20], friends and family [21], websites [22], online support groups [23], and medical professionals [24]. A study by Fox and Duggan [25] revealed that more than half of American adults use the Internet to obtain health information. Lam and Lam [26] have shown that many Australian residents over the age of 50 actively seek health-related information via the Internet. However, there is little information about health information-seeking behaviors and information sources used to obtain health information in the Middle Eastern [27] and developing countries [28]. Iran with a population of around 85 million people is one of these countries. Limited studies have addressed information seeking behavior of Iranian people [29]. A community-based cross-sectional study [30] that was conducted in Tehran (the capital of Iran) during two different periods: in August 2002 and 2010, showed that the most common sources of health information in 2002 were radio and television, caregivers, and Internet, and in 2010 radio and television, Internet and caregivers. A study by Baheiraei et al. [31] that was conducted in Tehran showed that mothers (51.11%) and same-sex friends (40.11%) were the preferred sources of health information for adolescents. Another study carried out in public libraries in Qazvin, Iran showed that the most common sources for seeking health information were “radio and television” and “discussions with others” [29].

During the past decade, most health information-seeking studies have been conducted in developed countries [27] in specific domains, while few studies have been conducted in Iran. Existing studies in Iran [29–33] have been conducted on a limited number of participants, in specific places (such as public libraries or universities), and for specific groups (such as women, mothers, or teenagers). These studies have reported different and scattered results based on their study context. On the other hand, people’s priorities may have changed over time. Hence, the results of these studies cannot be generalized to the whole population of this country. Today, in the patient-centered information approach, the active role of people in searching for information is emphasized, and it is necessary to identify people's information needs and understanding their concerns [8]. Initiatives such as self-management and self-care mostly relies on health information seeking and provision of information to people [34]. However, there is insufficient information about people's preferences and information needs while seeking health information and the tools they need. Nowadays, different information technology (IT) tools such as Internet search, computer, and mobile apps, and social networks are used to search for health information from different sources [35] and people receive health information based on their access to these tools. Studies have shown that new information technologies increase the levels of health-related knowledge [36], change health-related behaviors, and encourage people to visit physicians or seek health-related consultations [37]. The way health information is presented in these tools also affects the amount of health information gained by people and their knowledge level [38, 39]. Obtaining health information in each country depends on aspects such as age distribution, gender, cultural and educational factors, accessibility, intelligibility, and reliability of information sources [30, 40]. However, little information exists about the kind of IT tools preferred by Iranian people as a platform for health information seeking. In addition, to the best of our knowledge, no study on the national-level has investigated the preferred sources and IT tools used by Iranian to obtain health information. Therefore, this study was conducted to identify the sources Iranian people use to obtain health information and to determine and prioritize the preferred IT tools and the preferred method of presenting information in the IT tools.

## Methods

### Study design and population

This study was a national survey conducted from April to September 2021 in Iran. Based on the following formula [41] and considering the design effect of 1.5 and a 10% drop (to compensate for sample size attrition due to the elimination of incomplete questionnaires and to increase the accuracy of the results), a sample size of 4000 people (aged 18-75 years) was obtained.

$$n=\frac{{z}_{1-\frac{\alpha }{2}}^{2} p(1-p)}{{d}^{2}}$$

**Fig. 1:**
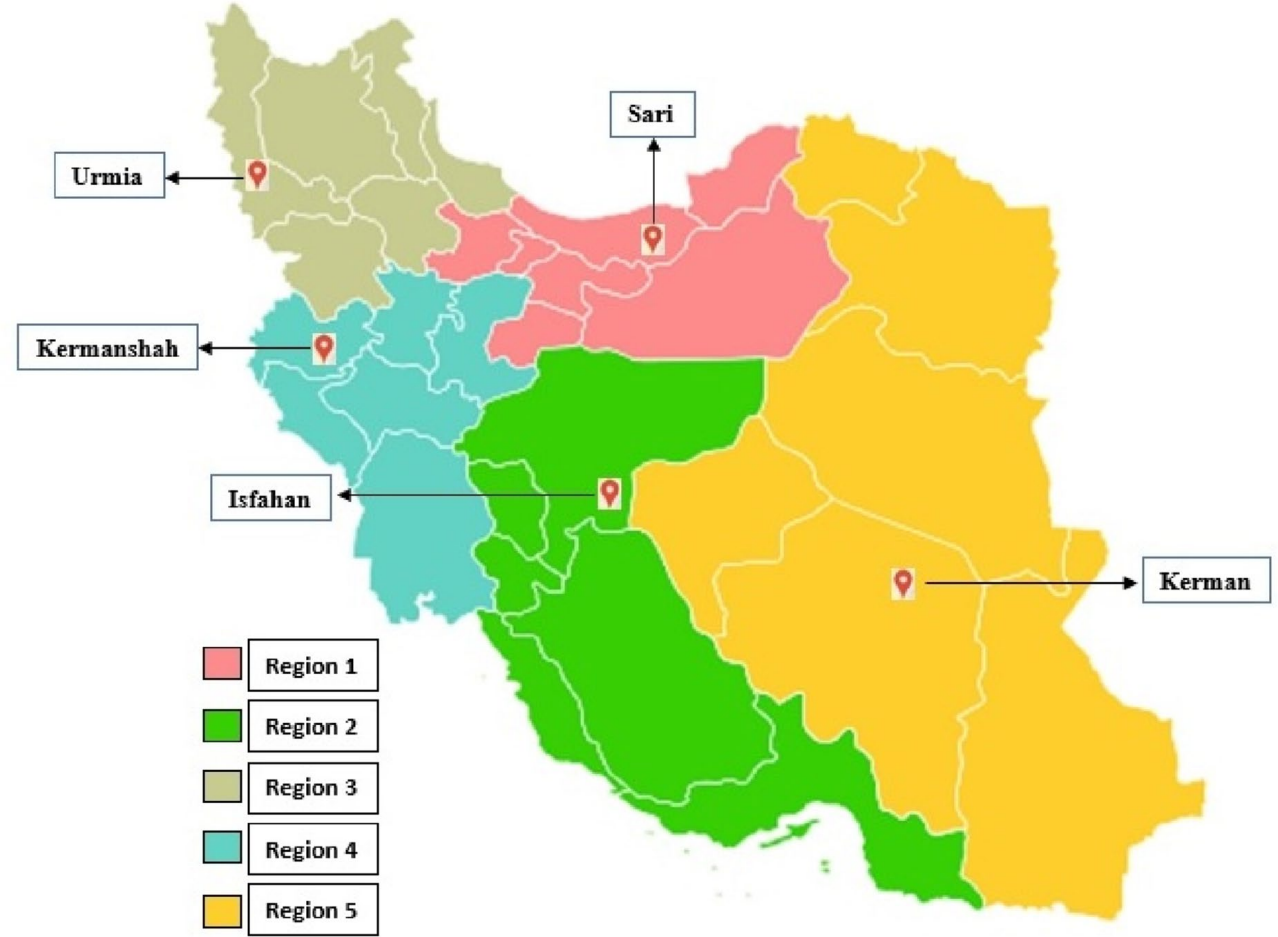
Regionalization of Iran's provinces (based on proximity factors, geographical location, and commonalities)

After calculating the sample size, multi-stage cluster sampling was used to select samples from the capital cities of different provinces of Iran. These cities were selected based on the regionalization of Iran's provinces (Fig. 1). According to this regionalization, Iran’s provinces are categorized according to the neighborhood, geographical location, and commonality factors in the form of 5 regions (Region 1: (Tehran, Qazvin, Mazandaran, Golestan, Alborz, and Qom), Region 2: (Isfahan, Fars, Bushehr, Chahar Mahal, and Bakhtiari, Semnan Hormozgan and Kohkiloyeh and Boyer Ahmad), Region 3: (East Azerbaijan, West Azerbaijan, Ardabil, Zanjan, Gilan and Kurdistan), Region 4: (Kermanshah, Ilam, Lorestan, Hamedan, Central, and Khuzestan), Region 5: (Khorasan Razavi, South Khorasan), North Khorasan, Kerman, Yazd and Sistan and Baluchistan) [42]. From each region, a province was randomly selected. Finally, the provinces of Mazandaran (region 1), Isfahan (region 2), West Azerbaijan (region 3), Kermanshah (region 4), and Kerman (region 5) were selected (Fig. 1). To distribute the questionnaires, the cluster sampling method was used in each selected city. For this purpose, in each of the capital cities of the selected provinces (the cities of Sari, Isfahan, Urmia, Kermanshah, and Kerman), different urban areas were identified. From each urban area, 16 neighborhoods, from each neighborhood 10 streets, and in each street one person was selected by simple random sampling. In this study, two criteria were used to include the participants: 1) all the people who were present at the place of distributing the questionnaires, and 2) voluntary participation. Also, the exclusion criteria for the participants were people under 18 years of age. Finally, from each capital city, the views of 800 people were examined.

### Data collection

The data were collected using a questionnaire developed (in Persian) based on experts' opinions and the review of related articles [43–46] (Additional file 1). Four medical informatics specialists confirmed the face and content validity of this questionnaire. The reliability of this questionnaire was confirmed by calculating Cronbach's coefficient alpha (α = 0.86). This questionnaire consisted of the following four Sects. (27 questions): 1- demographic information of the participants (e.g. gender, marital status, level of education, and chronic co-morbidity (5 questions)), 2- current sources of health information including individuals, printed materials or electronic sources (10 questions), 3- preferred IT tools for accessing health information including the Internet "websites", "mobile apps", "computer applications", "e-books", "social networks" and "computer games" (6 questions), and 4- the method of presenting health information in IT tools including the use of "texts", "images", "educational videos", "animations", "educational slides" and "audio files (podcasts)" (6 questions). In the questions related to the second to fourth parts of the questionnaire, a five-point Likert scale (1 = very little to 5 = very much) was used. Five questioning teams distributed the questionnaires. These teams had received the necessary face-to-face and distance training before the start of the study concerning how to distribute and complete the questionnaires. The questioning teams went to the selected streets of each city and invited the people in these areas to complete a paper questionnaire (one person from each selected street). Due to the spread of the Corona disease at the time of data collection, the link to the electronic questionnaire was also provided to the respondents through SMS or WhatsApp, as a replacement for the paper questionnaire for whom prefer to fill out the electronic questionnaire. The distribution of questionnaires in each city was continued until the calculated sample size was met.

### Statistical analyses

We used SPSS 24 and Stata 16 to analyze the data. The participants' answers to the questions were presented using frequency and percentage, and the mean and standard deviation of quantitative variables (such as age) were calculated. Before data analysis, the multiple imputation method was used for missing data. For this purpose, the answers given by the people to other questions were used to estimate the answers to the unanswered questions. To determine the priorities related to the questions of the second, third, and fourth parts of the questionnaire, the scores given to each question were calculated (1 = very low to 5 = very high). To calculate this score, first, the frequency of each Likert point was multiplied by the score of that point, and then these products were summed. Then, this sum was divided by the number of people who answered that question. Finally, the resulting score was a number between 1 and 5. Given that, we calculated the average scores given to each question (1 = very low to 5 = very high) to determine the questions' priorities, so we had a calculated score for each question (a numerical value). Therefore, we used the linear regression test to examine the relationship between qualitative variables (marital status, illness, gender, and education level) and questions from different parts of the questionnaire. Considering that the sample size was extracted equally from five capital cities (800 people in each city) and the population size of the cities was different, we used survey analysis in linear regression tests, and the ratio of the city population to the sample size in each city was used as a sampling weight in the analyses.

## Results

### Demographic information of the respondents

The average age of the participants was 36.45 ± 12.26. Most of the participants in this study were women (56%), people with bachelor's education (35%), and married (66.5%). Also, the majority of the participants (91%) were not suffering from any specific disease (Table 1).

**Table 1:** Demographic characteristics of participants

Demographic Information	n (%)
**Gender**	
Female	2235(55.9)
Male	1765(44.1)
**Marital status**	
Single	1342(33.5)
Married	2659(66.5)
**Education**	
Under the high school diploma	620(15.5)
High school diploma	1362(34.0)
Bachelor	1400(35.0)
Master’s degree and higher	619(15.5)
**Do you have a specific illness?**	
Yes	347(8.7)
No	3652(91.3)

### Information sources used to obtain health information

Most of the participants received health-related information through an "Internet search" (3.62), "consultation with family or friends" (3.43), using "social networks" (3.41), "searching specific websites" (3.41), and using "mobile apps" (3.27) (Table 2).

**Table 2:** Participants' use of information sources to obtain health information

Questions	Frequency					Percentage (by applying sampling weight)					Prioritization
	Very low	Low	Medium	High	Very high	Very low	Low	Medium	High	Very high	
Internet search	491	245	856	1089	1319	0.11	0.06	0.22	0.29	0.32	3.62
Consultation with family or friends	308	522	1100	1251	812	0.08	0.14	0.29	0.32	0.17	3.43
Using social networks	524	426	924	1119	1001	0.14	0.11	0.25	0.29	0.21	3.41
Searching in specific websites^a^	607	609	888	250	1602	0.18	0.18	0.24	0.08	0.32	3.41
Mobile applications	561	525	997	1075	827	0.14	0.14	0.27	0.29	0.16	3.27
Consultation with a physician or health care providers	665	534	1285	921	590	0.15	0.15	0.32	0.24	0.14	3.05
Radio or television	674	480	1326	975	536	0.16	0.15	0.33	0.24	0.12	3.05
Newspapers, magazines and other publications	1069	806	1153	632	329	0.26	0.22	0.29	0.16	0.07	2.58
E-books	1286	785	872	594	456	0.27	0.20	0.26	0.17	0.10	2.53
Printed books	1316	975	926	432	346	0.29	0.25	0.27	0.12	0.07	2.37

^a^This refers to websites that provide health information in public disease

The relationship between demographic information and sources of health information is shown in Table 3. To obtain health information, with each unit of increase in the average age of the participants, the use of "consulting with physicians or health service providers" was increased by 0.01 (*p* < 0.0001), "radio and television" by 0.01 (*p* < 0.0001), and "newspapers and magazines" by 0.004 (*p* = 0.027). Moreover, with one unit of increase in the average age of the participants, the use of "electronic books" decreased by 0.01 (*p* < 0.0001), "printed books" by 0.01 (*p* < 0.0001), "mobile apps" by 0.01 (*p* > 0.0001), "social networks" by 0.01 (*p* > 0.0001) and the "Internet search" by 0.02 (*p* > 0.0001).

**Table 3:** Relationship between demographic information and information sources for obtaining health information

Demographic Information	Participants’ use of information sources to obtain health information									
	**Consultation with a physician or health care providers**	**Consultation with family or friends**	**Radio or Television**	**Newspapers, magazines and other publications**	**Printed books**	**E-books**	**Internet search**	**Mobile applications**	**Using social networks**	**Searching in specific websites**
	**Regression Coefficients (95% CI)**									
**Age**	0.01 (0.007, 0.013)*	-0.001 (-0.002,0.0009)	0.01 (0.006,0.01)*	0.004 (0.0008,0.007)*	-0.01 (-0.013,-0.006)*	-0.01 (-0.013,-0.006)*	-0.02 (-0.023,-0.016)*	-0.01 (-0.013,-0.006)*	-0.01 (-0.013,-0.006)*	0.0004 (-0.003,0.004)
**Gender**										
Female	Ref, -	Ref, -	Ref, -	Ref, -	Ref, -	Ref, -	Ref, -	Ref, -	Ref, -	Ref, -
Male	-0.07 (-0.16,0.01)	-0.01 (-0.09,0.06)	0.07 (-0.01,0.16)	0.12 (0.03,0.21)*	0.01 (-0.07,0.10)	-0.14 (-0.24,-0.04)*	-0.04 (-0.14,0.04)	0.06 (-0.02,0.15)	0.11 (0.01,0.21)*	0.02 (-0.08,0.13)*
**Marital status**										
Single	Ref, -	Ref, -	Ref, -	Ref, -	Ref, -	Ref, -	Ref, -	Ref, -	Ref, -	Ref, -
Married	0.33 (0.23,0.42)*	-0.02 (-0.11,0.05)	0.31 (0.21,0.40)*	0.10 (0.01,0.20)*	-0.20 (-0.30,-0.11)*	-0.30 (-0.40,-0.19)*	-0.29 (-0.39,-0.20)*	-0.18 (-0.28,-0.09)*	-0.16 (-0.26,-0.06)*	0.05 (-0.06,0.17)
**Education**										
Under the high school diploma	Ref, -	Ref, -	Ref, -	Ref, -	Ref, -	Ref, -	Ref, -	Ref, -	Ref, -	Ref, -
High school diploma	-0.06 (-0.21,0.07)	-0.34 (-0.46,-0.22)*	-0.31 (-0.46,-0.17)*	0.03 (-0.11,0.18)	0.06 (-0.07,0.20)	0.09 (-0.06,0.25)	0.17 (0.01,0.33)*	0.31 (0.16,0.46)*	0.22 (0.05,0.38)*	0.12 (-0.06,0.30)
Bachelor	-0.05 (-0.20,0.08)	-0.38 (-0.50,-0.26)*	-0.45 (-0.59,-0.31)*	0.03 (-0.10,0.18)	0.24 (0.10,0.38)*	0.26 (0.11,0.42)*	0.40 (0.25,0.55)*	0.16 (0.01,0.31)*	0.23 (0.06,0.39)*	-0.23 (-0.41,-0.06)*
Master’s degree and higher	0.10 (-0.06,0.26)	-0.29 (-0.42,-0.15)*	-0.52 (-0.69,-0.36)*	0.08 (-0.09,0.25)	0.24 (0.08,0.40)*	0.43 (0.25,0.61)*	0.60 (0.43,0.77)*	0.13 (-0.03,0.31)	0.33 (0.15,0.51)*	-0.21 (-0.41,-0.01)*
**Do you have a specific illness?**										
No	Ref, -	Ref, -	Ref, -	Ref, -	Ref, -	Ref, -	Ref, -	Ref, -	Ref, -	Ref, -
Yes	0.15 (-0.03,0.33)	0.01 (-0.15,0.17)	0.06 (-0.13,0.25)	-0.28 (-0.44,-0.11)*	-0.26 (-0.43,-0.09)*	-0.28 (-0.47,-0.10)*	-0.60 (-0.81,-0.40)*	-0.64 (-0.83,-0.45)*	-0.61 (-0.80,-0.42)*	0.26 (0.02,0.49)*

*Ref* Indicate Reference, *CI* Indicate Confidence Intervals

^*^P-Value < 0.05

On average obtaining health information from "family or friends", and "radio and television" was higher in the people with an education level under the high school diploma than the people with other education levels (*p* < 0.0001). The average use of "printed books", "e-books", and "social networks" was higher in the people with academic education than the people with an education level under a high school diploma (Table 3). The people with education under a high school diploma used the "Internet search" method to obtain health information less than the people with other education levels. The average use of "mobile apps" by the participants was 0.31 higher among those who had a high school diploma than those who had an education level lower than a high school diploma (*p* < 0.0001). The average use of "mobile apps" by the participants was 0.16 higher among those who had associate's and bachelor's degrees than those who had a degree lower than a high school diploma (*p* = 0.034). The average use of "specific websites" by the participants was higher among people who had a degree lower than a high school diploma than the people with an academic education. The average use of "newspapers", "magazines", and other publications and "social networks" to obtain information was higher for men compared to women. The average use of "electronic books" to obtain information was lower in men than in women (Table 3).

The average use of "newspapers", "magazines", and other publications (B = 0.28, *p* = 0.001), "printed books" (B = 0.26, *p* = 0.002), "electronic books" (B = -0.28, *p* = 0.003), the "Internet search" (B = -0.60, *p* > 0.0001), "mobile apps" (B = -0.64, *p* > 0.0001) and "social networks" (B = -0.61, *p* > 0.0001) to obtain health information in participants who did not have an underlying disease were lower than in the participants who had an underlying disease (Table 3). On average, the participants who did not have an underlying disease searched "specific websites" to obtain health information 0.26 times more than the participants who had an underlying disease (*p* = 0.029). The average use of "printed books" (B = 0.20, *p* > 0.0001), "e-books" (B = 0.30, *p* > 0.0001), "Internet search" (B = 0.29, (*p* > 0.0001), "mobile apps" (B = 0.18, *p* > 0.0001) and "social networks" (B = 0.16, *p* = 0.002) by married people was lower than their use by unmarried people. The average use of "consultation with physicians or health service providers" (B = 0.33, *p* < 0.0001), "radio and television" (B = 0.31, *p* < 0.0001)) and "newspapers", "magazines", and other publications (B = 0.10, *p* = 0.023) were higher in married people (Table 3).

### Tendency to use different electronic tools to receive health information

The participants preferred "social networks" (3.67), "website" (3.56), and "mobile app" (3.50), respectively, as the most suitable tools for receiving health information (Table 4).

**Table 4:** Tendency to use different electronic tools to receive health information and different methods of presenting health information

Questions	Frequency					Percentage (by applying sampling weight)					Prioritization
	Very low	Low	Medium	High	Very high	Very low	Low	Medium	High	Very high	
**Tendency to use different electronic tools to receive health information**											
Social networks	325	420	790	1111	1305	0.09	0.11	0.20	0.28	0.32	3.67
Website	264	424	1179	976	1109	0.08	0.09	0.28	0.26	0.29	3.56
Mobile application	315	455	1054	1178	960	0.09	0.11	0.27	0.31	0.22	3.50
Electronic book (e-book)	759	1039	1018	562	586	0.18	0.24	0.28	0.16	0.14	2.79
Computer application	852	1052	1050	582	455	0.24	0.27	0.26	0.14	0.09	2.68
Computer games	1326	924	822	454	435	0.34	0.24	0.22	0.11	0.09	2.43
**Tendency to use different methods of presenting health information**											
Image	113	314	902	1345	1293	0.04	0.07	0.25	0.36	0.28	3.85
Educational videos	246	440	876	1125	1281	0.08	0.10	0.21	0.30	0.31	3.69
Text	237	435	1220	1121	953	0.07	0.10	0.30	0.30	0.23	3.53
Educational slides	479	687	1009	889	891	0.13	0.16	0.26	0.24	0.21	3.25
Animation	523	734	975	857	879	0.15	0.18	0.23	0.24	0.20	3.21
Audio file (podcast)	620	713	1004	808	808	0.12	0.18	0.28	0.22	0.20	3.11

The relationship between demographic variables and preferred electronic tools for receiving health information is shown in Table 5. For one unit increase in age, the average preference of the participants to receive health information decreased by 0.01 for "websites", "mobile apps", "computer applications", "electronic books", and "social networks" (*p* > 0/0001) and by 0.007 for "computer games" (*p* > 0.003) (Table 5). People with education under the high school diploma were less willing to receive health information through "websites", "mobile apps", "computer applications", and "e-books" compared to people with an academic degree (Table 5).

**Table 5:** Relationship between demographic information and tendency to use different electronic tools to receive health information and different methods of presenting health information

Demographic Information	Tendency to use different electronic tools to receive health information						Tendency to use different methods of presenting health information					
	**Website**	**Mobile application**	**Computer application**	**Electronic book**	**Social networks**	**Computer games**	**Text**	**Image**	**Educational videos**	**Animation**	**Educational slides**	**Audio file (podcast)**
	**Regression Coefficients (95% CI), *p*-value**											
**Age**	**-0.01** (-0.013,-0.006)*	**-0.01** (-0.013,-0.006)*	**-0.01** (-0.013,-0.006)*	**-0.01** (-0.013,-0.006)*	**-0.01** (-0.013,-0.006)*	**-0.007** (-0.01,-0.003)*	**-0.005** (-0.008,-0.001)*	**-0.007** (-0.008,-0.005)*	**-0.004** (-0.007,-0.0008)*	**-0.003** **(-0.006,-0.0009)**	**-0.009** (-0.01,-0.005)*	**-0.005** (-0.008,-0.001)*
**Gender**												
Female	**Ref, -**	**Ref, -**	**Ref, -**	**Ref, -**	**Ref, -**	**Ref, -**	**Ref, -**	**Ref, -**	**Ref, -**	**Ref, -**	**Ref, -**	**Ref, -**
Male	**0.01** **(-0.07,0.09)**	**0.15** (0.06,0.24)*	**0.19** (0.10,0.29)*	**0.02** **(-0.07,0.11)**	**0.05** **(-0.04,0.15)**	**0.25** (0.16,0.35)*	**0.001** **(-0.08,0.08)**	**0.04** **(-0.02,0.12)**	**0.02** **(-0.06,0.10)**	**0.10** **(0.007,0.20)**	**-0.05** **(-0.14,0.03)**	**-0.57** **(-0.14,0.03)**
**Marital status**												
Single	**Ref, -**	**Ref, -**	**Ref, -**	**Ref, -**	**Ref, -**	**Ref, -**	**Ref, -**	**Ref, -**	**Ref, -**	**Ref, -**	**Ref, -**	**Ref, -**
Married	**-0.15** (-0.24,-0.05)*	**-0.07** **(-0.16,0.02)**	**-0.18** (-0.28,-0.08)*	**-0.33** (-0.43,-0.23)*	**-0.02** **(-0.13,0.07)**	**-0.25** (-0.35,-0.15)*	**-0.12** (-0.21,-0.04)*	**-0.19** (-0.27,-0.12)*	**-0.14** (-0.23,-0.05)*	**-0.12** (-0.22,-0.02)*	**-0.21** (-0.31,-0.12)*	**-0.04** **(-0.14,0.04)**
**Education**												
Under the high school diploma	**Ref, -**	**Ref, -**	**Ref, -**	**Ref, -**	**Ref, -**	**Ref, -**	**Ref, -**	**Ref, -**	**Ref, -**	**Ref, -**	**Ref, -**	**Ref, -**
High school diploma	**0.10** **(-0.03,0.25)**	**0.19** (0.05,0.34)*	**0.23** (0.08,0.38)*	**0.04** **(-0.11,0.20)**	**0.08** **(-0.07,0.23)**	**0.003** **(-0.14,0.15)**	**0.11** **(-0.02,0.25)**	**0.001** **(-0.11,0.11)**	**-0.07** **(-0.20,0.06)**	**-0.16** (-0.32,-0.01)*	**0.04** **(-0.10,0.19)**	**-0.16** (-0.31,-0.01)*
Bachelor	**0.19** (0.05,0.33)*	**0.16** (0.02,0.31)*	**0.19** (0.04,0.34)*	**0.20** (0.04,0.35)*	**0.007** **(-0.14,0.16)**	**-0.003** **(-0.15,0.14)**	**0.08** **(-0.04,0.22)**	**-0.01** **(-0.12,0.10)**	**0.02** **(-0.10,0.15)**	**-0.01** **(-0.16,0.13)**	**0.07** **(-0.06,0.22)**	**-0.001** **(-0.14,0.14)**
Master’s degree and higher	**0.18** (0.01,0.34)*	**0.12** **(-0.03,0.29)**	**0.23** (0.06,0.41)*	**0.36** (0.19,0.54)*	**0.13** **(-0.03,0.31)**	**0.03** **(-0.13,0.20)**	**0.06** **(-0.09,0.22)**	**0.13** **(-0.0009,0.26)**	**0.27** (0.11,0.42)*	**0.33** (0.15,0.50)*	**0.37** (0.20,0.54)*	**0.16** (0.002,0.33)*
**Do you have a specific illness?**												
No	**Ref, -**	**Ref, -**	**Ref, -**	**Ref, -**	**Ref, -**	**Ref, -**	**Ref, -**	**Ref, -**	**Ref, -**	**Ref, -**	**Ref, -**	**Ref, -**
Yes	**-0.32** (-0.51,-0.13)*	**-0.49** (-0.69,-0.30)*	**-0.34** (-0.51,-0.17)*	**-0.27** (-0.45,-0.10)*	**-0.5** (-0.70,-0.31)*	**-0.01** **(-0.19,0.16)**	**-0.07** **(-0.23,0.09)**	**-0.18** (-0.35,-0.005)*	**0.12** **(-0.03,0.29)**	**-0.08** **(-0.29,0.12)**	**-0.17** **(-0.37,0.03)**	**0.05** **(-0.12,0.23)**

*Ref* Indicate Reference, *CI* Indicate Confidence Intervals

^*^ *P*-Value < 0

Compared to women, men had a higher willingness to receive health information through "mobile apps" (B = 0.15), "computer applications" (B = 0.19), and "computer games" (B = 0.25) (*p* ≤ 0.0001). The rate of receiving health information through the "website" (B = 0.32), "mobile apps" (B = 0.49), "computer applications" (B = 0.34), "e-books" (B = 0.27), and "social networks" (B = 0.50) was lower in the participants who did not have an underlying disease than the participants who had an underlying disease (*p* ≤ 0.002). Married people sought less information through "websites" (B = 0.15), "computer applications" (B = 0.18), "electronic books" (B = 0.33) and "computer games" (B = 0.25) than single participants (*p* ≤ 0.001) (Table 5).

### Tendency to use different methods of presenting health information

The details of different methods of presenting health information are shown in Table 4. In order of priority, the participants preferred presenting health information in the form of "image" (3.85), "educational video" (3.69), and "text" (3.53) (Table 4). The relationship between demographic variables and different methods of presenting health information is shown in Table 5.

As shown in Table 5, a one-unit increase in age, decreases the preference of the participants to receive health information in the form of "text" (B = -0.005), "image" (B = -0.007), "educational video" (B = -0.004), "educational slide" (B = -009) and "audio file" (B = -005) (*p* ≤ 023). The average willingness of the participants to receive health information through educational "videos", "animations", and "educational slides" was lower in people with education under the high school diploma than in people with an academic education (Table 5). Compared to women, the average willingness of men to receive health information through animation was 0.10 higher (*p* = 0.034). The preference of the participants who did not have an underlying disease to receive health information through image was 0.18 lower than the participants who had an underlying disease (*p* = 0.043). On average, the married participants had a lower willingness to receive health information in the form of "text" (B = 0.12), "image" (B = 0.19), "educational video" (B = 0.14), "animation" (B = 0.12) and "educational slides" (B = 0.21) than the single participants (*p* ≤ 0.01) (Table 5).

## Discussion

In the current study, the Internet was reported as the first source of obtaining health information. A study by Alishahi-Tabriz et al. [30] showed that the "Internet", alongside "radio and television", is one of the primary sources of obtaining health information for Iranian people. Another study [29] in Iran showed that the most common source for seeking health information was "radio and television". Contrary to our result, the results of these two studies in Iran show that over a decade, the "Internet" has become the main source of obtaining health information for people, and they are more active information seekers than passive ones. One of the reasons for the priority of the "Internet" over other tools for obtaining information is its significant growth and use among Iranian people in recent years [47]. Another reason can be the difficulty of accessing health care providers and their reluctance in responding to people's informational needs. In line with this result, previous studies in other countries also identified the "Internet" as the main source for seeking health information [13–15, 48, 49]. Various studies have shown that about three-quarters of American people search the Internet to obtain health information [50, 51].

"Family or friends" were the second source of obtaining health information. "Family and friends" often act as informal sources for obtaining health information [52] and sometimes even act as surrogates seeking information for others [53]. The reason for this can be the availability of family members or friends, and intimacy and trust in this group. In this regard, Smith's study [54] showed that "healthcare professionals" consistently have the highest level of trust among people, however, the level of trust in other sources of information such as "family", "friends", the "Internet", and "television" is also relatively high.

Other sources of obtaining health information in our study were "social networks", Internet "websites", and "mobile apps". In the study of Weiner et al. [55], after the "Internet", other sources of health information were "family", "health care professionals", "websites", and "friends". Also, in the study of Jaks et al. [14], the highly used sources for obtaining information by parents were the "Internet", "social media", "mobile apps", "websites", and "social networks (chats and posts) ", respectively. In our study, "physicians and healthcare providers" were not among the main priorities of participants for receiving health information. Contrary to this result, other studies [16, 44, 54, 56] in other countries reported that "physicians and healthcare providers" are among the main sources of health information. One of the reasons for this difference in the results can be the period of conducting and publishing previous studies. These studies have been conducted in the last ten years, and today, with the advances in information technology, the preferred sources of obtaining health information have changed. A reason for the difference between the results of the present study and other studies can be the variation in the payment systems among societies. In per-patient payment health systems [57], physicians may spend little time on each patient. Another reason could be the lack of adequate communication skills between patients and physicians. Patients may search online sources for health information to avoid disruption to the work of healthcare providers [58]. Likewise, cultural differences may affect the choices of information sources. In this regard, Das et al. [59] showed that the relationship between health-seeking behavior and diverse gender elements, such as gendered social status, social control, ideology, gender process, marital status, and procreative status changes across settings. Also, Lee's study [45] showed that according to their cultural background, American mothers prefer human sources (such as "physicians", "nurses", "spouses", and "other relatives"), while Korean immigrant mothers prefer non-human sources (such as "online communities", and "books").

Consistent with the previous studies [60–62], in the current study, traditional sources ("radio or television", "magazines", "newspapers", "electronic" and "printed books") had the least priority. Since these sources do not fully meet the growing needs of people for health information [63], people prefer human sources to non-human sources [64–66]. A study [29] surveying 200 people in public libraries in one of the cities of Iran reported that "television" and "conversation with people" are the most frequent sources of health information. Also, another study [30] in Tehran (the capital of Iran) at two-time points in 2002 and 2010 showed that the most common sources of health information in 2002 were "radio and television", "caregivers", and the "Internet", and in 2010 were "radio and television", the "Internet" and "caregivers". According to the results of these two studies [29, 30] that were carried out on limited populations in Iran, "radio and television" was among the primary sources of health information for Iranian people. Contrary to this result, our study showed that this source is no longer among Iranian people's main sources of health information. This inconsistency could be due to the low number of participants in these studies and the difference in study setting and year of study. Also, this trend shows the change in people's preferences over time in Iran and other countries. In this regard, Morschhauser’s study [67] showed that the most common sources of information in 2003 for cancer patients in America were the "Internet", "books", and "health care providers", but in 2007, "books" were the most used sources and health care providers were the least cited sources.

In the present study, with increasing age, the participant's willingness to seek information from "health care providers", "radio", "television", "newspapers", and "magazines" raised, and from "e-books", "printed books", "mobile apps", "social networks", and the "Internet" decreased. Different studies [62, 68] have also shown that compared to younger people, older people receive health information mostly from "newspapers", "magazines", "television", and "health care professionals" and less from the "Internet". Based on the results, people with higher education mostly obtain information from "printed books", "e-books", and "social networks", while people with lower education obtain it from "family or friends", "radio", and "television". Also, a study [30] in Tehran (the capital of Iran) also showed that highly educated people use the "Internet" more, and low-educated people use "friends and family" or "radio and television" to access health information. Studies in Iran [29] and other countries [7, 69–71] show that younger and higher educated people use web sources more than older and lower educated users. In line with our results, Kutner et al. [72] showed that people with lower literacy levels were less likely to use written sources (such as "books", "magazines", and "newspapers"), and more likely to use "radio and television" to obtain health information. A systematic review study also showed [73] that people with low health literacy tend to get information from physicians or television instead of written materials. In our study, men mostly use "newspapers", "magazines", and "social networks", and women use "e-books" to obtain health information. Various studies have shown that people's gender affects the information-seeking process and attitude of people toward it [74, 75]. Other studies [62, 76] have also shown that women and those with a higher level of education are more likely to access information through the "Internet" and "books". Women are active searchers for health information and show more attention and sensitivity to their health and the implementation of preventive measures than men [77, 78]. Contrary to our result, a study by Gavgani [29] in Iran showed no significant statistical relationship between the gender of people and sources of health information. It can be due to the small sample size and the specific study setting (public libraries). Our results showed that participants with an underlying (chronic) disease mostly search for disease-related health information in "newspapers", "magazines", "printed books" and "e-books", the "Internet", and "social networks". In this regard, Ayers and Kronenfeld [79] revealed that people having a higher number of chronic diseases use the "Internet" more than people with healthier conditions do. Also, other studies have shown that people with co-morbidities search the "Internet" more than healthy people [80, 81]. Morschauser [67] also showed that the most common sources of primary information for cancer patients are the "Internet", "books", and "health care providers".

In the present study, most of the participants preferred to receive health information through "social networks". In line with this result, in the studies of Kim et al. [66] and DiSalvo et al. [82], participants preferred to use "social networks" to receive health-related information. In general, the expansion of social networks has influenced the way people obtain health information [66]. For example, people who have wide access to "social networks" have healthier behavioral habits due to better access to related health information [66]. Internet "websites" were the second preferred tool for receiving health-related information. The results of a review study [83] also showed that most of the participants use the Internet "websites" to get health information. Currently, with the increasing growth of the volume of information, the number of websites that provide health information to users is increasing [83, 84]. Obtaining health information can result in self-confidence in personal health management and a higher level of health literacy. On the other hand, information is available on the "Internet" 24 h a day, and people are not exposed to judgments or questions when searching for personal and confidential health issues that people avoid declaring in face-to-face visits. Another important point is that the "Internet" can provide the possibility of creating interaction and receiving emotional support for people [83, 84].

From the point of view of the participants, "images", "educational videos", and "text" were the most preferred methods for displaying health-related information. In line with these results, the study of Nam and Kim [38] showed that people could easier identify and recall health content that is supported with "images" than the content provided only in the form of written "text". In the study of Frisch et al. [39], the participants who were exposed to multimedia presentation (both "text" and "image") had a higher amount of health information than the participants who were exposed to only textual information.

In the present study, age, gender, education level, marital status, and the history of comorbidities have a significant effect on people's willingness to receive health information from various tools such as Internet "websites", "mobile apps", "computer applications", and "e-books". Based on Demirci et al.'s [85] results, age, gender, education level, place of residence, and the frequency of the "Internet" affect the participants' health information search behavior on the "Internet". In addition, in the study of Link et al. [86], age, gender, education, and income were identified as influential factors. In the present study, the average willingness to receive health information through "mobile apps", "computer applications", and "computer games" was higher in men than in women. Also, no significant difference was observed between men and women in receiving health information through Internet "websites". Contrary to this result, in Demirci et al.'s [85] study, women tended to search for health information on the "Internet" more than men. The inconsistency in these results can be related to the difference in the country and culture of the participants. Our research was conducted in Iran, but Demirci et al.'s [85] research was conducted in Turkey.

In our study, the mean scores of the preferred sources were numerically very close to each other (Table 2), which can indicate the importance of all these sources in seeking health information and the possibility of searching for information in a combination of using these sources. According to the channel complementarity theory [87], a theoretical framework, people are motivated to obtain information about a topic using all possible sources. According to the supplementary version of this theory presented by Ruppel and Raine [46], sources that have each of these four characteristics, including "convenience level", "anonymity", "adaptability", and "access to medical expertise" while seeking health information, are used more often. Therefore, it can be concluded that people do not use only one specific source (such as the Internet) in seeking health information, but different sources may be used. In this regard, two studies [88, 89] have shown that people often use multiple information sources to obtain information to learn or deal with a health condition.

Overall, the findings of our study show that the selection of health information sources and tools can be affected by various factors such as demographic factors, culture, time, and personal characteristics of people. Also, our study indicates that the preferred sources of Iranian people for seeking health information became similar to that observed in developed countries, where people use different sources alongside IT tools to obtain health information. Hence, health policymakers should consider these factors to meet users' needs. Given the results of this study, future research is required to investigate the preferred sources and IT tools for seeking health information over time. To our knowledge, this was the first study that identified the preferred sources of health information at the national level in Iran. Meanwhile, it is the first study that identified the preferred IT tools and the way of presenting health information in these tools. Various studies have investigated the sources of health information in recent years in different societies, but limited studies have been conducted at the national level on a sufficient number of participants.

## Limitations of the study

This study had three limitations. First, in this study, due to the large number of questions and the time constraints because of the Corona pandemic, we could not investigate the reasons and obstacles of choosing different sources. However, by analyzing the relationship between preferred sources and demographic factors we determined the effect of these factors on the selection of sources. Future studies can examine the reasons and obstacles for choosing different sources.of health information in different societies. Second, due to varied cultural factors and social characteristics, the generalization of the results of this study to other societies should be done with caution. Third, our questionnaire did not contain demographic variables such as place of residence (urban and rural), occupation, income level, computer literacy level, and race, which can be important predictive factors in health information-seeking patterns from different sources. Future studies can examine the possible relationship between these factors and health information sources.

## Implications of the study

The results of this study can help health policymakers and planners in countries with similar situations or cultural affinities such as Middle Eastern and developing countries to better plan for providing health information in these societies. The priorities identified in this study can be used in the development of health programs and policies to meet the health information needs of the people. Policymakers should invest in the development of the Internet and its infrastructure to improve people's access to the required health information. Based on the results, providing health information to patients and their relatives and friends can enhance their awareness and disease management. The results of this study can also help the developers of these technologies to understand the preference of people concerning IT tools and the preferred way of presenting health information in these tools and to develop user-centered information systems. Policymakers and governments can increase people's access to health information by developing preferred IT tools such as specific "social networks", "websites", and "mobile applications". We suggest that Developers provide health information through people's preferred methods ("image", "video", and "text") in IT tools to improve users' acceptance. The results of examining the relationship between demographic factors and information sources and IT tools in this study, help online health providers, the private sector, planners, and policymakers to tailor their services to the information needs of people. For example, since older people, people with low education, or married people prefer traditional sources (such as "physicians", "radio" and "television", and "magazines") to obtain health information, it is recommended to provide the required information of this groups through "medical consultations" in clinics or "radio and television" programs, "magazines" or similar media. Younger people or people with academic education prefer newer sources (such as "e-books", "Internet searches", "social networks", and "mobile applications"). Therefore, governments should also sufficiently invest on these platforms. Most men prefer "newspapers" and "social networks", and women prefer "e-books" to obtain health information in Iran. Hence, gender preferences should be taken into account when planning for the interventions addressing health information needs of individuals. By providing health information in the form of "text", "images", "videos", or "educational slides", policymakers can respond to the needs of younger, single, or academically educated people. Also, providing health information in the form of animation to men and images for patients with chronic co-morbidity can help them. However, due to factors such as the advancement of IT tools and methods, gradual changes of people preferences and variation of preferences among different communities, all information-based intervention should be tailored to users’ priorities and preferences.

## Conclusions

In this study, the sources of obtaining health information, the preferred IT tools, and the methods of presenting health information in these tools were identified at a national level. The results of this study showed that most Iranian people obtain health information through " Internet search", "consulting with family or friends", using "social networks", searching on "specific websites" and using "mobile apps”, respectively. In order of priority, people prefer to obtain information from "social networks", "websites" and "mobile apps" and receive information in the form of "images", "educational videos" and use of the “text" in these tools. Comparing the results of this study and previous studies reveal that Iranian people are more active information seekers than passive ones compared to a decade ago. In our study, participants’ average rating score of health information sources was numerically very close to each other. Our findings suggest that a combination of different sources can meet health information needs of different groups of individuals. Hence, health policymakers and governments should invest on developing all possible information sources. Also, the results showed that most demographic factors (age, gender, marital status, education level, and co-morbidities) influence the way of choosing health information sources. Providing information to patients through appropriate sources can improve their trust in the healthcare system, reduce confusion in the use of information, and promote their awareness before consulting with physicians. Consequently, they can make good communication with healthcare providers and ask relevant questions resulting in a high rate of compliance with physicians' recommendations. Future studies can investigate the reasons and barriers that lead to choosing different sources of health information. Healthcare policymakers and providers as well as developers of IT tools can use the results of this study to plan and develop policies that promote access to health information according to people's preferences and socio-demographics characteristics.

### Supplementary Information

**Additional file 1.**

## Data Availability

The datasets used and/or analyzed during the current study are available from the corresponding author upon a reasonable request.
