# Supplementary material for: The preferred IT sources and tools of Iranian people for accessing health information
Source: BMC Public Health. 2023 Oct 12;23:1986. doi: 10.1186/s12889-023-16334-y (PMC10568770; doi:10.1186/s12889-023-16334-y)
Supplement: Supplementary file 1 — Additional file 1. [file 12889_2023_16334_MOESM1_ESM.docx]

**Dear respondent**

This questionnaire is designed to identify the sources you use to obtain health information and determine and prioritize your preferred information technology (IT) tools and method of presenting information in the IT tools. Answering these questions accurately will increase the accuracy of this research. There is no need to mention the name and surname to answer. The collected data will remain confidential. Thank you in advance for your cooperation.

**1- Demographic Information**

**1- Gender :** Female Man

**2- Age :** ………..……… years old (for example : *35 years old*)

**3- Marital Status :** Single Married

**4- Education :** Under the high school diploma High school diploma Bachelor

Master’s degree and higher

**5- Do you have a specific illness (or chronic co-morbidity)?** Yes No

**2- Information resources to obtain health information**

- **To what extent do you currently use the following information sources to obtain health information?**

(Please tick ✓)

| **Information resources** |  |  |  |  |  |
| --- | --- | --- | --- | --- | --- |
|  | **Very High** | **High** | **Medium** | **Low** | **Very Low** |
| Internet search  (*For example, search in Yahoo or Google, etc.*) |  |  |  |  |  |
| Consultation with family or friends |  |  |  |  |  |
| Using social networks  (*For example, Facebook, YouTube, WhatsApp,*  *Instagram, etc.*) |  |  |  |  |  |
| Searching in specific websites |  |  |  |  |  |
| Mobile applications |  |  |  |  |  |
| Consultation with a physician or health care  providers |  |  |  |  |  |
| Radio or television |  |  |  |  |  |
| Newspapers, magazines and other publications |  |  |  |  |  |
| E-books |  |  |  |  |  |
| Printed books |  |  |  |  |  |

**3- Preferred** **Information Technology (IT) tools for accessing health information**

- **To what extent do you consider each of the following Information Technology (IT) tools suitable for obtaining information electronically?** (Please tick ✓)

| **Information Technology (IT) tools** |  |  |  |  |  |
| --- | --- | --- | --- | --- | --- |
|  | **Very High** | **High** | **Medium** | **Low** | **Very Low** |
| Social networks |  |  |  |  |  |
| Website |  |  |  |  |  |
| Mobile application |  |  |  |  |  |
| Electronic book (e-book) |  |  |  |  |  |
| Computer application |  |  |  |  |  |
| Computer games |  |  |  |  |  |

**4- The method of presenting health information in Information Technology (IT) tools**

- **To what extent do you consider each of the following methods suitable for presenting information in IT tools?** (Please tick ✓)

| **The method of presenting information** |  |  |  |  |  |
| --- | --- | --- | --- | --- | --- |
|  | **Very High** | **High** | **Medium** | **Low** | **Very Low** |
| Images |  |  |  |  |  |
| Educational videos |  |  |  |  |  |
| Text |  |  |  |  |  |
| Educational slides |  |  |  |  |  |
| Animation |  |  |  |  |  |
| Audio file (podcast) |  |  |  |  |  |
